# Complete chloroplast genome of vulnerable medicinal plant *Saraca asoca* (Fabaceae)

**DOI:** 10.1080/23802359.2020.1715300

**Published:** 2020-01-21

**Authors:** Mohammad Ajmal Ali, Soo-Yong Kim, Tapan Kumar Pan, F. Al-Hemaid, M. S. Elshikh, Meena Elangbam, Joongku Lee, Mohammad Abul Farah, Khalid M. Al-Anazi

**Affiliations:** a Department of Botany and Microbiology, College of Science, King Saud University, Riyadh, Saudi Arabia;; b International Biological Material Research Center, Korea Research Institute of Bioscience and Biotechnology, Daejeon, Republic of Korea;; c University Department of Botany, Tilka Manji Bhagalpur University, Bhagalpur, India;; d Genetics Laboratory, Centre of Advanced Studies in Life Sciences, Manipur University, Canchipur, India;; e Department of Environment and Forest Resources, Chungnam National University, Daejeon, Republic of Korea;; f Genetics Laboratory, Department of Zoology, College of Science, King Saud University, Riyadh, Saudi Arabia

**Keywords:** Chloroplast genome, vulnerable, medicinal plant, *Saraca asoca*, Fabaceae, adulterants, *rbcL*

## Abstract

The complete chloroplast genome sequences of vulnerable medicinal plant *Saraca asoca* (Roxb.) Willd. (Fabaceae) was sequenced. A total of 5,206,216,851 paired-end filtered reads of 151 bp were obtained. The plastome length (including LSC, SSC, IRa, and IRb) was 137,743 bp (GC content: 35.26%). A total of 126 coding genes which includes 97 CDS, 24 tRNA, and five rRNA genes were annotated. The phylogenetic analysis attempts to establish molecular signature in order to differentiate genuine sample of *S. asoca* from its adulterants easily.

*Saraca asoca* (Roxb.) Willd. (family Fabaceae, sub family Detarioideae), a rain-forest tree, native to Indian subcontinent, is one of the highly traded medicinal tree whose bark greatly valued for treatment of gynecological disorders (Singh et al. 2015). The over exploitation due to increasing commercial demand of the crude drug material mainly of its bark have resulted to vulnerable in wild (IUCN 2011); therefore, being extensively adulterated with *Bauhinia variegata* L., *Mesua ferrea* L., *Polyalthia longifolia* (Sonn.) Thwaites*, Shorea robusta* Gaertn., and *Trema orientalis* (L.) Blume in herbal raw drug trading (Hegde et al. 2018). An attempt to assess the extent of adulteration in the raw herbal trade of *S. asoca* using DNA barcoding was validated by NMR spectroscopic techniques (Urumarudappa et al. 2016). *rbcL*-ISSR based DNA barcodes (Hegde et al. 2018) are least user-friendly. The gradual development in NGS platforms and bioinformatics tools for the data analyses have revolutionized our understanding about comparative genomics, chloroplast biology, intracellular gene transfer, conservation biology, diversity, phylogeny, and also facilitated in the enhancement of the plant agronomic traits and to produce high-value agricultural products through genetic engineering.

The chloroplast DNA was extracted from the leaves [Voucher: ‘MAA & TKPAN-116’ (BHAG)] collected from botanical garden (25°14′25″N 86°56′55″E), TMBU, Bhagalpur, India, and were sequenced at Illumina sequencing platform. A total of 5,206,216,851 paired-end filtered reads of 151 bp were obtained which were assembled using spades (Bankevich et al. 2012). A total of 126 coding genes including 97 protein-coding, 24 tRNA, and five rRNA genes were annotated using GeSeq (Tillich et al. 2017) and tRNAsacn-SE (Lowe and Eddy 1997). The total plastome length (Genbank accession number: MN866115) was 137,743 bp (GC content: 35.26%). The phylogenetic tree from the set of the GenBank accession number (see the phylogenetic tree) of *rbcL*[-the chloroplast gene proposed for plant DNA barcoding (CBOL 2009)] sequence of *S. asoca* and its adulterated with *B. variegata*, *M. ferrea*, *P. longifolia, S. robusta,* and *T. orientalis* using MEGA X (Kumar et al. 2018) revealed promising potential to be used as molecular authentication to discriminate genuine sample from adulterants (Figure 1).

**Figure 1.**
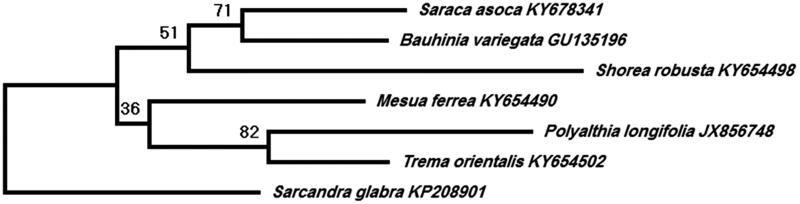
The maximum-likelihood (ML) tree based on *rbcL* gene from a total number of seven representative species of Rosids.
